# Dynamic prediction for clinically relevant pancreatic fistula: a novel prediction model for laparoscopic pancreaticoduodenectomy

**DOI:** 10.1186/s12893-020-00968-5

**Published:** 2021-01-04

**Authors:** Runwen Liu, Yunqiang Cai, He Cai, Yajia Lan, Lingwei Meng, Yongbin Li, Bing Peng

**Affiliations:** 1 West China Clinical Medicine Academy, Sichuan University, Chengdu, China; 2 Department of Pancreatic Surgery, West China Hospital, Sichuan University, No. 37, Guoxue Alley, Chengdu, 610041 Sichuan Province China; 3 Department of General Surgery, Chengdu Shangjin Nanfu Hospital, Chengdu, China; 4 West China School of Public Health, SCU, Chengdu, China

**Keywords:** Dynamic prediction, C-reactive protein to albumin ratio, Clinically relevant postoperative pancreatic fistula, Drainage fluid amylase, Laparoscopic pancreaticoduodenectomy

## Abstract

**Background:**

With the recent emerge of dynamic prediction model on the use of diabetes, cardiovascular diseases and renal failure, and its advantage of providing timely predicted results according to the fluctuation of the condition of the patients, we aim to develop a dynamic prediction model with its corresponding risk assessment chart for clinically relevant postoperative pancreatic fistula after laparoscopic pancreaticoduodenectomy by combining baseline factors and postoperative time-relevant drainage fluid amylase level and C-reactive protein-to-albumin ratio.

**Methods:**

We collected data of 251 patients undergoing LPD at West China Hospital of Sichuan University from January 2016 to April 2019. We extracted preoperative and intraoperative baseline factors and time-window of postoperative drainage fluid amylase and C-reactive protein-to-albumin ratio relevant to clinically relevant pancreatic fistula by performing univariate and multivariate analyses, developing a time-relevant logistic model with the evaluation of its discrimination ability. We also established a risk assessment chart in each time-point.

**Results:**

The proportion of the patients who developed clinically relevant postoperative pancreatic fistula after laparoscopic pancreaticoduodenectomy was 7.6% (19/251); preoperative albumin and creatine levels, as well as drainage fluid amylase and C-reactive protein-to-albumin ratio on postoperative days 2, 3, and 5, were the independent risk factors for clinically relevant postoperative pancreatic fistula. The cut-off points of the prediction value of each time-relevant logistic model were 14.0% (sensitivity: 81.9%, specificity: 86.5%), 8.3% (sensitivity: 85.7%, specificity: 79.1%), and 7.4% (sensitivity: 76.9%, specificity: 85.9%) on postoperative days 2, 3, and 5, respectively, the area under the receiver operating characteristic curve was 0.866 (95% CI 0.737–0.996), 0.896 (95% CI 0.814–0.978), and 0.888 (95% CI 0.806–0.971), respectively.

**Conclusions:**

The dynamic prediction model for clinically relevant postoperative pancreatic fistula has a good to very good discriminative ability and predictive accuracy. Patients whose predictive values were above 14.0%, 8.3%, and 7.5% on postoperative days 2, 3, and 5 would be very likely to develop clinically relevant postoperative pancreatic fistula after laparoscopic pancreaticoduodenectomy.

## Background

Despite the advent of laparoscopic pancreaticoduodenectomy (LPD) in 1992 [1] and its advantages, such as shortened surgical incisions, reduced hospital stay, and improved long-term prognosis [2–4], the occurrence rate of clinically relevant postoperative pancreatic fistula (CR-POPF), which includes grade B and C with a demand of clinical intervention, remains as high as 10–34% [5]. Moreover, CR-POPF can cause abdominal infection, postoperative bleeding, and even death [6, 7]. Recent studies have conventionally used some of the indexes that are constant to predict the occurrence of POPF in a static way [8–10]. Among them, postoperative drainage fluid amylase (DFA) and C-reactive protein-to-serum-albumin ratio (CAR) seemed to be the accurate and widely used indexes for the prediction of CR-POPF, especially in a particular time-point, in which they are suggested to be useful and critical predictive indexes [11–14]. However, preoperative and intraoperative factors indicating CR-POPF seemed to vary [15–18], furthermore, such prediction result cannot be updated according to the changes in the patient’s fluctuating state and examination results over time.

Recently, dynamic prediction model that is based on a time-relevant factor and patients’ condition has been used in predicting the occurrence of diabetes, cardiovascular diseases and renal failure [19–21], which shows the relationship between the fluctuation of the predictive indexes over time and the risk of adverse events, providing timely predictions according to the changes in the condition of the patients [22, 23]. Given the fluctuation of the pathological and physiological conditions contributes to the occurrence of CR-POPF, leading to the relevant turbulence of examiner results of the patient [24, 25], such model for CR-POPF is thus needed.

## Methods

### Aim, design and setting of the study

We aimed to develop a dynamic prediction model for CR-POPF by combining baseline factors and time-relevant variables, including postoperative DFA and CAR, within postoperative day 5.

The development of model is processed by (1) sifting viable variables by univariant analysis, (2) define feasible landmark time points, (3) find out cut-off points of doable factors relevant to CR-POPF, (4) establish time-relevant multi-variant Logistics model, (5) assessment of discriminative ability of model on each landmark time points, (6) establish abridged assessment table of the risk of CR-POPF calculated by regression model.

### Patients and definition of CR-POPF

In this study, we retrospectively reviewed the data from all the patients who underwent LPD at West China Hospital between January 2016 and April 2019 (n = 284). Patients who were converted to open surgery due to: (1) intraoperatively uncontrollable hemorrhage, (2) the tumor size was too large to expose clearly (> 5 cm), (3) the vascular anastomosis was too difficult or difficult to carry out safely under laparoscopy, were excluded (n = 33). Therefore, there were 251 cases eventually enrolled in this study.

The outcome, CR-POPF, was defined in accordance with the updated 2016 International Study Group of Pancreatic Surgery (ISGPS) consensus guidelines [26].

### Clinical variables

We retrospectively reviewed the following baseline variables obtained from the patients’ electronic medical records:

0 Basic information: age, sex, body mass index (BMI), smoking status, alcohol consumption, abdominal surgical history, hypertension, and diabetes mellitus;
1 Preoperative hematologic test: hemoglobin, platelet, white blood cells, neutrophil, lymphocyte, monocyte, red blood cells, hematocrit, blood type, total bilirubin, direct bilirubin, indirect bilirubin, aspartate transaminase, alanine transaminase, total protein, albumin, blood urea nitrogen, creatinine, glucose, triglyceride, cholesterol;
2 Surgical relevant information: American Society of Anesthesiologists (ASA) grading; operative time, preoperative biliary drainage, intraoperative blood loss, dissection and reconstruction of vessels, texture of pancreas, and chief pancreatic duct’s diameter. Blood examinations were performed within 1 week prior to LPD.
3 Time-relevant postoperative data: DFA, C-reactive protein (CRP), and albumin levels on postoperative days (PODs) 1, 2, 3, and 5. The CAR was calculated by dividing the serum CRP level by the peripheral serum albumin level.

### Statistical analysis

To extract the factors relevant to the occurrence of CR-POPF and to define the time-window of postoperative drainage fluid amylase and CAR related to the occurrence of CR-POPF, we tested for differences between patients with and without CR-POPF by using chi-squared tests for categorial variables and independent t-tests or Mann–Whitney U rank-sum tests for continuous variables depending on their distribution. The latter were expressed as either $\stackrel{-}{x}\pm s$ or $M({Q}_{25}, {Q}_{75})$ in accordance with statistical method. The preoperative and intraoperative variables with a *P* value < 0.10, along with postoperative DFA and CAR at time points where *P* values were less than 0.10, were included in the multi-variable analysis. Among the selected variables and their corresponding time-point, the continuous variables were assigned 0 and 1 if their values were less than or no less than their cut-off point on their corresponding receiver operating characteristic (ROC) curve, which means that all the significant variables for the dynamic prediction model were categorized.

To establish the dynamic prediction model, each time-relevant logistics regression model in each suitable time-point was made for all selected baseline variables combined with time-relevant variables in the corresponding time-point after binarization according to their cut-off points [19]. The *P* value of all the variables in the multi-variable analysis should be less than 0.05. After establishing the formula of the predictive value according to the regression model and the formula $p=\frac{exp({\beta }_{0}+{\beta }_{0}{X}_{1}+\dots +{\beta }_{n}{\beta }_{n})}{1+exp({\beta }_{0}+{\beta }_{0}{X}_{1}+\dots +{\beta }_{n}{\beta }_{n})}$, we used the area under the receiver operating characteristic curve (AUC) to evaluate the model’s discriminative ability at each time point and determined the cut-off point of the predictive value outputted by the model on ROC at each time point.

In addition, we established a risk assessment chart for CR-POPF by combining all risk factors in each time-point in the prediction model and presenting a predictive value of each corresponding logistic regression model of each time point. To distinguish the different risk levels of each circumstance shown in the chart, we used the color key changing from blue to white and to red gradually, with the increasing likelihood of the occurrence of CR-POPF on each cell. Moreover, we highlighted the cells with scores above their corresponding cut-off point by using red dotted circles.

All statistical analyses were performed using SPSS Statistics for Windows version 23.0 (IBM Corp., Armonk, NY). The risk assessment chart was completed using Microsoft® Excel® 2019 MSO (16.0.12430.20112) 32-bit (Microsoft Corp., Redmond, USA). The 95% confidence intervals (CI) were also calculated. The statistical methods and results of this study were reviewed by a Medical statistician (Yajia Lan, PhD) at Sichuan University.

## Results

**Fig. 1:**
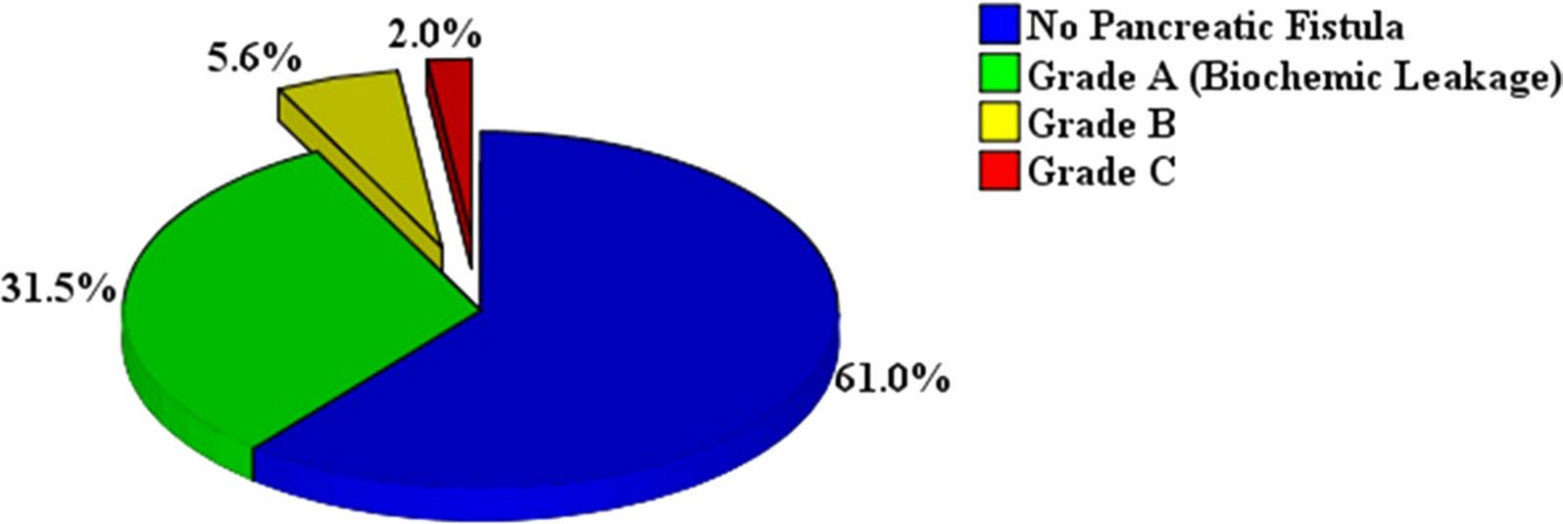
The Proportion of Patients of The Grade of Postoperative Pancreatic Fistula. According to the updated 2016 International Study Group of Pancreatic Surgery (ISGPS) consensus guidelines, pancreatic fistula grade B and C are defined as clinically relevant Postoperative Pancreatic Fistula

As shown in Fig. 1, POPF grade A (biochemical leak) occurred in 31.5% (79/251) of the cases, whereas CR-POPF occurred in 7.6% (19/251) of the patients, with grades B and C occurring in 5.6% (14/251) and 2.0% (5/251) of patients, respectively.

Table 1 presents the results of the univariate analysis of baseline data. Among the baseline data, preoperative albumin (patients with CR-POPF 35.95 ± 5.07 g/L vs patients without CR-POPF 38.65 ± 5.29 g/L, *P* = 0.033), creatine (patients with CR-POPF 80.68 ± 30.13 μmol/L vs patients without CR-POPF 67.88 ± 25.40 μmol/L, *P* = 0.038), and dissection and reconstruction of vessels (patients with CR-POPF 0/19 vs patients without CR-POPF 37/195, *P* = 0.086) were the preoperative and intraoperative factors associated with the occurrence of CR-POPF (*P* < 0.10). Among the time-relevant variables shown in Table 2, in the univariate analysis, DFA and CAR on PODs 2, 3, and 5 were associated with the occurrence of CR-POPF (*P* < 0.10), with median and 95% CI of DFA of the patients with or without CR-POPF in each time-point were 1149 (653–2651) vs 630 (137–2013), 978 (415–4990) vs 248 (56–934), and 454 (178–3604) vs 63 (20–240), respectively, and those of CAR were 6.36 (3.71–8.30) vs 4.84 (3.27–6.17), 6.44 (4.20–9.51) vs 4.57 (3.12–6.14), and 3.78 (2.4–5.79) vs 2.62 (1.37–3.72), respectively. The binarization of the continuous variables for CR-POPF is shown in Table 3. The cut-off points of albumin and creatine levels were 38.1 g/L and 64 μmol/L, respectively. The cut-off points of DFA and CAR were 642 IU/L and 5.88 on POD 2, 409 IU/L and 7.69 on POD 3, and 162 IU/L and 3.37 on POD 5.

**Table 1:** Univariate analysis of the baseline factors

Factors	Patients without CR-POPF	Patients with CR-POPF	*P* value
Operation age (years)	59.88 ± 12.62	64.21 ± 11.49	0.149
Gender			0.726
Male	137	12	
Female	95	7	
Body mass index, kg/m^2^	21.47 ± 4.15	23.03 ± 6.33	0.132
Smoking^@^			0.210
Yes	80	9	
No	147	9	
Drinking^@^			0.381
Yes	66	7	
No	161	11	
History of hypertension^@^			0.500*
Yes	35	4	
No	192	14	
History of diabetes^@^			0.146#
Yes	34	0	
No	193	18	
Hemoglobin, g/L	123.47 ± 20.51	120.21 ± 18.71	0.504
Platelet, 10^9^/L	217.41 ± 88.23	213.79 ± 84.79	0.863
White blood cells, 10^9^/L	5.90 ± 2.40	6.12 ± 2.83	0.719
Neutrophil, 10^9^/L	3.97 ± 2.17	4.20 ± 2.59	0.676
Lymphocyte, 10^9^/L	1.34 ± 0.54	1.28 ± 0.54	0.649
Monocyte, 10^9^/L	0.46 ± 0.21	0.47 ± 0.18	0.882
Red blood cells, 10^12^/L	4.03 ± 0.61	3.82 ± 0.59	0.134
Hematocrit	0.36 ± 0.05	0.35 ± 0.05	0.434
Blood type^@^			0.943^#^
A	76	6	
B	46	4	
AB	23	1	
O	82	7	
Total bilirubin, μmol/L	30.2 (11.4–180.0)	19.8 (10.7–187.2)	0.818
Direct bilirubin, μmol/L	18.2 (4.5–152.5)	10.7 (4.6–149.3)	0.801
Indirect bilirubin, μmol/L	8.9 (5.4–19.0)	8.4 (5.4–26.4)	0.892
Asparate transaminase, IU/L	48 (20–124)	57 (18–127)	0.903
Alanine transaminase, IU/L	54 (17–173)	38 (17–273)	0.958
Total protein, g/L	63.09 ± 8.62	60.65 ± 5.23	0.225
Albumin, g/L	38.65 ± 5.29	35.95 ± 5.07	**0.033**
Blood urea nitrogen, mmol/L	4.5 (3.6–5.6)	4.8 (3.8–6.7)	0.333
Creatinine, μmol/L	67.88 ± 25.40	80.68 ± 30.13	**0.038**
Glucose, mmol/L	5.75 ± 1.94	5.51 ± 1.34	0.591
Triglyceride, mmol/L	1.80 ± 0.91	1.97 ± 1.07	0.428
Cholesterol, mmol/L	4.85 ± 2.07	4.86 ± 2.13	0.976
ASA grading			0.858#
1	37	3	
2	118	11	
3 or above	77	5	
Operation time, min	345 (292—412)	358 (296—419)	1.000
Preoperative biliary drainage			1.000*
Yes	49	4	
No	183	15	
Intraoperative blood loss, mL	100 (100–200)	100 (50–138)	0.182
Dissection and reconstruction of vessels			0.086*
Yes	37	0	
No	195	19	
Texture of pancreas			0.130
Hard	139	8	
Soft	93	11	
Pancreatic Duct’s diameter, cm	0.4(0.3–0.5)	0.4 (0.3–0.4)	0.357

Variables with* P* < 0.10 (in bold) were to be included in the next multivariate analysis

*ASA* American Society of Anesthesiologists

^*^Fisher Exact test were used

^#^Monte Caro Exact test were used

^@^n = 245

**Table 2:** Univariate analysis of drainage fluid amylase and C-reactive protein to albumin ratio in postoperative days

Landmark time point	Variables	Patients without CR-POPF	Patients with CR-POPF	*P* value
POD1	Drainage fluid amylase, IU/L	1224 (195–2482)	1082 (700–2143)	0.533
	CAR	3.16 (2.31–4.19)	3.15 (0.52–7.23)	0.916
POD2	Drainage fluid amylase, IU/L	630 (137––2013)	1149 (653–2651)	**0.041**
	CAR	4.84 (3.27–6.17)	6.36 (3.71–8.30)	**0.055**
POD3	Drainage fluid amylase, IU/L	248 (56–934)	978 (415–4990)	** < 0.001**
	CAR	4.57 (3.12––6.14)	6.44 (4.20–9.51)	**0.024**
POD5	Drainage fluid amylase, IU/L	63 (20–240)	454 (178–3604)	** < 0.001**
	CAR	2.62 (1.37–3.72)	3.78 (2.46–5.79)	**0.027**

Variables with* P* < 0.10 (in bold) were to be included in the next multivariate analysis

*POD* postoperative day, *CAR* C-reactive protein to albumin ratio, *AUC* area under curve, *ROC* receiver operator characteristic curve

**Table 3:** Receiver operating characteristic analysis and cut-off points for variants relevant to CR-POPF

Time	Variables	AUC of ROC (95%CI)	Cut-off point	*P* value
Preoperative	Albumin	0.637 (0.518—0.757)	38.1 g/L	0.046
	Creatine	0.656 (0.543—0.769)	64 μmol/L	0.024
POD2	DFA	0.642 (0.540—0.744)	642 IU/L	0.041
	CAR	0.674 (0.488—0.859)	5.88	0.055
POD3	DFA	0.749 (0.651—0.847)	409 IU/L	< 0.001
	CAR	0.681 (0.541—0.821)	7.69	0.024
POD5	DFA	0.769 (0.644—0.895)	162 IU/L	< 0.001
	CAR	0.679 (0.539—0.820)	3.37	0.027

*CR-POPF* clinically relevant pancreatic fistula, *DFA* Drainage fluid amylase, *CAR* C-reactive protein to albumin ratio

**Fig. 2:**
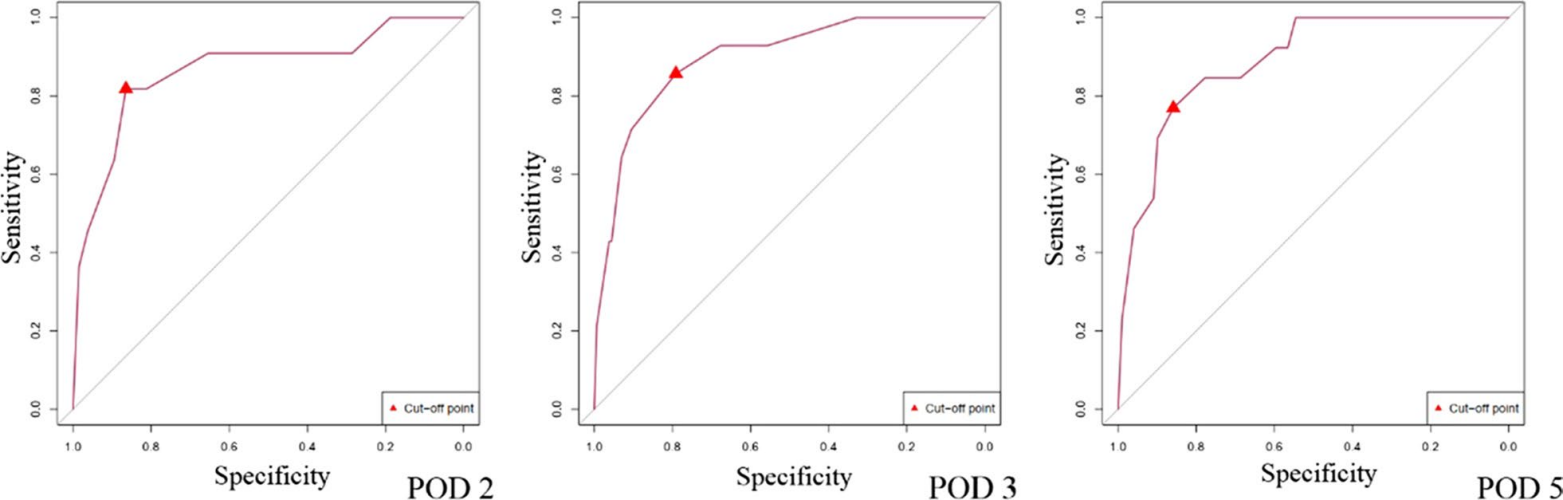
Receiver Operating Characteristic Curve of The Prediction Model on Each Landmark Time-point. In *POD 2*, AUC = 0.866 (95% CI 0.737–0.996, *P* < 0.001, cut-off point: 14.0%, Sensitivity: 81.9%, specificity: 86.5%); In *POD 3*, AUC = 0.896 (95% CI 0.814–0.978, *P* < 0.001, cut-off point: 8.3%. sensitivity: 85.7%, specificity: 79.1%); and In *POD 5*, AUC = 0.888 (95% CI 0.806–0.971, *P* < 0.001, cut-off point: 7.4%, sensitivity: 76.9%, specificity: 85.9%). *AUC* area under curve, *POD* postoperative day, *CI* coefficient interval

After binarizing the preoperative albumin and creatine levels and postoperative DFA and CAR on PODs 2, 3, and 5 according to the receiver operating characteristic analysis and theirs cut-off points, we established the logistic model of the preoperative albumin and creatine levels, dissection and reconstruction of vessels, and both of the time-relevant variables on PODs 2, 3, and 5. The time-relevant logistic models on PODs 2, 3, and 5 for CR-POPF are shown in Table 4. Given that the dissection and reconstruction of the vessel was not an independent factor of the occurrence of CR-POPF, the models in all of the time points only consisted of preoperative albumin and creatine levels and the two postoperative time-relevant variables, which were all independent factors found to be associated with the occurrence of CR-POPF on PODs 2, 3, and 5 (*P* < 0.05). Among these factors, albumin level > 38.1 g/L was the protective factor for CR-POPF (hazards ratio (HR): 0.169 (0.037–0.766), *P* = 0.021 on POD 2, HR: 0.152 (0.037–0.619), *P* = 0.009 on POD 3, HR: 0.138 (0.029–0.665), *P* = 0.014 on POD 5). Creatine level > 64 μmol/L had the highest HR among all the factors [HR: 27.884 (2.825–275.269)]. According to all of the time-relevant logistic regression models, the prediction value of the patients developing CR-POPF were $P=\frac{exp(-5.726-1.780[Alb\ge 38.1g/L]+1.810[Cr\ge 64\mu mol/L]+2.839[DFA\ge 642IU/L]+1.496 [CAR\ge 5.88])}{1+exp(-5.726-1.780[Alb\ge 38.1g/L]+1.810[Cr\ge 64\mu mol/L]+2.839[DFA\ge 642IU/L]+1.496 [CAR\ge 5.88])}$ on POD 2, $P=\frac{exp(-5.714-1.887[Alb\ge 38.1g/L]+3.328[C\ge 64\mu mol/L]+1.856[DFA\ge 409IU/L]+1.815 [CAR\ge 7.69])}{1+exp(-5.714-1.887[Alb\ge 38.1g/L]+3.328[C\ge 64\mu mol/L]+1.856[DFA\ge 409IU/L]+1.815 [CAR\ge 7.69])}$ on POD3 and $P=\frac{exp(-4.992-1.979[Alb\ge 38.1g/L]+2.465[C\ge 64\mu mol/L]+2.002[DFA\ge 162IU/L]+1.967 [CAR\ge 3.37])}{1+exp(-4.992-1.979[Alb\ge 38.1g/L]+2.465[C\ge 64\mu mol/L]+2.002[DFA\ge 162IU/L]+1.967 [CAR\ge 3.37])}$ on POD 5, of whichcorresponding cut-off points were 14.0% (sensitivity: 81.9%, specificity: 86.5%), 8.3% (sensitivity: 85.7%, specificity: 79.1%), and 7.4% (sensitivity: 76.9%, specificity: 85.9%) (Fig. 2), respectively. The models in all the time points had good to very good discrimination ability, with AUC on PODs 2, 3, and 5 of 0.866 (0.737–0.996), 0.896 (0.814–0.978), and 0.888 (0.806–0.971), respectively.

**Table 4:** Multi-variate logistic regression analysis on each landmark time point

Landmark time point	Variables	B	S.D	HR (95%CI)	*P* value
POD2	Alb	− 1.780	0.772		
	< 38.1 g/L			Ref	
	≥ 38.1 g/L			0.169 (0.037–0.766)	0.021
	Cr	1.810	0.903		
	< 64 μmol/L			Ref	
	≥ 64 μmol/L			6.110 (1.041–35.869)	0.045
	DFA	2.839	1.145		
	< 642 IU/L			Ref	
	≥ 642 IU/L			17.100 (1.812–161.401)	0.013
	CAR	1.496	0.761		
	< 5.88			Ref	
	≥ 5.88			4.465 (1.004–19.855)	0.049
	Constant	− 5.726	1.422	–	< 0.001
POD3	Alb	− 1.887	0.718		
	< 38.1 g/L			Ref	
	≥ 38.1 g/L			0.152 (0.037–0.619)	0.009
	Cr	3.328	1.168		
	< 64 μmol/L			Ref	
	≥ 64 μmol/L			27.884 (2.825–275.269)	0.004
	DFA	1.856	0.757		
	< 409 IU/L			Ref	
	≥ 409 IU/L			6.397 (1.450–28.219)	0.014
	CAR	1.815	0.736		
	< 7.69			Ref	
	≥ 7.69			6.141 (1.451–25.592)	0.014
	Constant	− 5.714	1.275	–	< 0.001
POD5	Alb	− 1.979	0.802		
	< 38.1 g/L			Ref	
	≥ 38.1 g/L			0.138 (0.029–0.665)	0.014
	Cr	2.465	0.980		
	< 64 μmol/L			Ref	
	≥ 64 μmol/L			11.767 (1.724–80.322)	0.012
	DFA	2.002	0.783		
	< 162 IU/L			Ref	
	≥ 162 IU/L			7.400 (1.595–34.345)	0.011
	CAR	1.967	0.795		
	< 3.37			Ref	
	≥ 3.37			7.151 (1.505–33.976)	0.013
	Constant	− 4.992	1.223	–	< 0.001

*S. D.* standard division, *HR* hazardous ratio, *Alb* albumin, *Cr* creatine, *POD* postoperative day, *CAR* C-reactive protein to albumin ratio

**Fig. 3:**
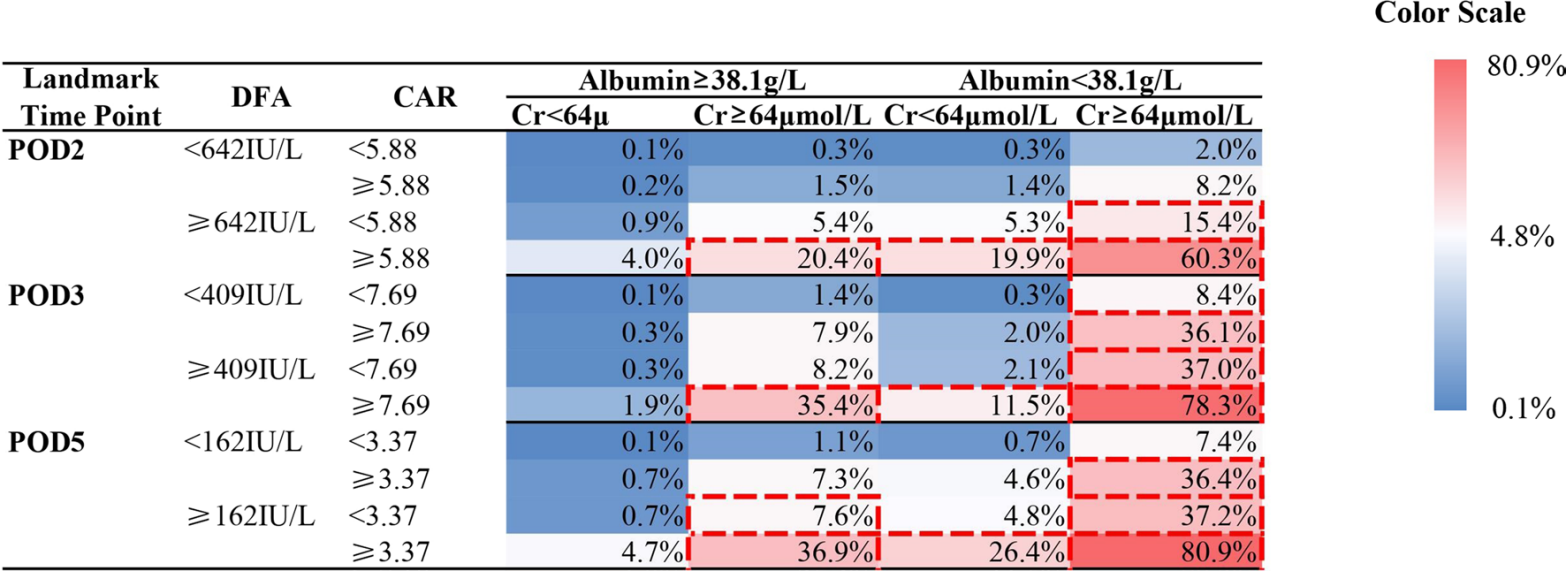
The Risk of CR-POPF Calculated by Regression Model on Each Landmark Time Point. The cells circled by red borders represented that theirs risk were higher than cut-off points in theirs corresponding time points. *POD* postoperative day, *CAR* C-reactive protein to albumin ratio, *Cr* creatine

Figure 3 shows the risk assessment chart for CR-POPF. The figure in each cell of each chart represents the predictive score of CR-POPF on PODs 2, 3, and 5. Among the patients who underwent LPD, the patients with lower preoperative albumin level, higher preoperative creatine level, and higher postoperative DFA and CAR obtained the highest predictive score (60.3%, 78.4%, and 80.9% on PODs 2, 3, and 5 respectively), which outclassed the other cases presented in the chart.

## Discussion

Our result demonstrates that combining preoperative and intraoperative static data with time-relevant postoperative variables to dynamically predict CR-POPF has good to very good discriminative ability and predictive accuracy. By combining preoperative albumin and creatine levels with postoperative DFA and CAR on PODs 2, 3 and 5, the predictive ability of our model has a better performance and a higher accuracy than the traditional static model, including the fistula risk score (FRS), as well as the followed-up modified alternate FRS (a-FRS) and ultra-alternate FRS (ua-FRS), which were all conducted not so accurate enough on cohorts including Asian groups (AUC < 0.7) [18, 27, 28].

Furthermore, the traditional prediction models for POPF or CR-POPF provide results that stay constant regardless of the patients’ fluctuating state over time. Developing a dynamic prediction model for CR-POPF will provide us a prediction result that could be rectify according to the fluctuations of the examination results and patients’ conditions, especially after the patients experienced visceral dissection and reconstruction as well as loss and recovery of visceral function [1, 29], when the homeostasis of the cases fluctuates rapidly [24, 25]. And, it could offer us timely predictions of the patients’ risk of developing of CR-POPF, which could contribute to making personalized treatment decisions with less deviation [30]. In addition, the model presented in this study is the first one to dynamically predict CR-POPF with a corresponding risk assessment chart, due to its reflection of the relationship between the turbulence of the risk score within in time window and the occurrence of an event, the relationship between time, and pathological state would be taken into account [22, 23].

It has proven that DFA is the reliable index that could predict the occurrence of CR-POPF. Several reports have shown that high postoperative DFA is an independent risk factor associated with CR-POPF [12–14, 31]. CAR, calculated by dividing the serum CRP level by the serum albumin level, which means dividing the positive and negative acute-phase reactants [32–34], reflects the acute inflammatory intensity [35], incorporating individual differences. As mentioned in a recent report, CAR was also an independent predictive indicator of CR-POPF [11]. Our research has shown that, except for POD 1, DFA and CAR on PODs 2–5 predict the occurrence of CR-POPF. The possible reason why DFA and CAR on POD1 cannot predict the occurrence of CR-POPF is that the level of CRP usually peaks at 48–72 h in response to inflammation [36], and the portion of the cases with biochemical leak with a relatively high DFA on POD 1 was large [37].

Amongst all the static factors, our research has also shown that the hypoalbuminemia leads to a higher risk of developing CR-POPF. As reported previously, albumin is a negative acute-phase reactant and its low level is associated with inflammation severity, disease prognosis, and mortality [33, 34, 38]. In terms of nutritious status, it has also been reported that hypoalbuminemia would indicate a low baseline nutrition status [38]. The low nutritional status leads to a higher risk of developing CR-POPF [39]. Combining the abovementioned factors, the occurrence of the CR-POPF is probably associated with the high level of inflammation and relatively poor nutritional state of a patient. Moreover, our result showing that a high preoperative creatine level contributes to a higher risk of CR-POPF is support by the finding of a previous study reporting that preoperative asymptomatic renal dysfunction is an independent risk factor for CR-POPF after PD and is associated with increased complications after various of types of surgery [40–43].

For most preoperative and intraoperative static indexes, our result shows that only a few of them could predict the occurrence of CR-POPF, apart from preoperative serum albumin and creatine levels. This is possibly due to the fact that there’s still no strong consensus about the association between these factors and CR-POPF, which probably leads to the inconsistencies in the prediction models developed recently [9, 10, 16, 18]. However, we noted that the patients who developed CR-POPF tend to have an advanced age [44, 45], higher BMI [46, 47], softer pancreas [48–50], and smaller pancreatic ducts [48, 51–55], were less likely to have diabetes [47, 56], and were more likely to have had portal vein resection [57], which are comparable to findings of previous research.

For the prevention and mitigation of the occurrence of CR-POPF, apart from preoperative evaluation by routinely blood examines, computer tomography, and intraoperative methods [58], postoperative prophylactic methods should also been taken on patients with higher risk of CR-POPF. First of all, on the basis of focusing on the volume, color and biochemical examine results of their abdominal drainage, somatostatin such as octreotide would be considered into administration to inhibit trypsin secretion in accordance to the patients’ situation [59], where antibiotics would be administrated if there are signs of infection.[60]. And, the effectiveness of preventive application of pasiresotide and negative pressure drainage to reduce the incidence of pancreatic fistula, have been proved effective in recent reports respectively [61, 62]. In addition, other reports also show the effectiveness of some treatments to postoperatively mitigate pancreatic fistula, such as hydrocortisone, and so-called triple drug therapy (TDT, including gabexate mesilate, octreotide, and carbapenem antibiotics) [63, 64]. The recent existence of postoperative measures has made the result of the prediction model more helpful.

There are some limitations of our study. First, this is a retrospective study with a limited number of cases, which could generate a retrospective bias and insufficient for setting a validation cohort. Second, the model would be complicated to be easily applied without the assistance of risk assignment chart, since it is focused on the prediction of CR-POPF in specific time period. Third, this study only developed a dynamic prediction of CR-POPF after LPD by DFA and CAR. In the future, the prediction of pancreatic fistula by using other dynamic indicators and its external multi-center validation should be included in further studies.

## Conclusion

The dynamic prediction model for CR-POPF has a good to very good discriminative ability and predictive accuracy. Patients whose predictive values were above 14.0%, 8.3%, and 7.5% on PODs 2, 3, and 5 would be very likely to develop CR-POPF after LPD.

## Data Availability

The data generated and analyzed during the current study came from the electronic medical record (EMR) system of West China Hospital of Sichuan University. Due to the data usage policy of West China College of Medicine and West China Hospital of Sichuan University, such data is not publicly available but are available from the corresponding author on reasonable request.

## References

[1] Gagner M, Pomp A (1994). Laparoscopic pylorus-preserving pancreatoduodenectomy. Surg Endosc.

[2] Choi M, Hwang HK, Rho SY, Lee WJ, Kang CM (2019). Comparing laparoscopic and open pancreaticoduodenectomy in patients with pancreatic head cancer: oncologic outcomes and inflammatory scores. J Hepato-Biliary-Pancreatic Sci.

[3] El Nakeeb A, Attia M, El Sorogy M, Ezzat H, Shehta A, Salem A (2019). Laparoscopic pancreaticodudenectomy for periampullary tumor: should it be a routine? A propensity score-matched study. Surg Laparosc Endosc Percut Tech.

[4] Zhou W, Jin W, Wang D, Lu C, Xu X, Zhang R (2019). Laparoscopic versus open pancreaticoduodenectomy for pancreatic ductal adenocarcinoma: a propensity score matching analysis. Cancer Commun.

[5] Pulvirenti A, Ramera M, Bassi CJTG (2017). Modifications in the International Study Group for Pancreatic Surgery (ISGPS) definition of postoperative pancreatic fistula. Hepatology.

[6] Van Buren G, Bloomston M, Hughes SJ, Winter J, Behrman SW, Zyromski NJ (2014). A randomized prospective multicenter trial of pancreaticoduodenectomy with and without routine intraperitoneal drainage. Ann Surg..

[7] Darnis B, Lebeau R, Chopin-Laly X, Adham M (2013). Postpancreatectomy hemorrhage (PPH): predictors and management from a prospective database. Langenbecks Arch Surg.

[8] Dalla Valle R, De Bellis M, Pedrazzi G, Lamecchi L, Bianchi G, Pellegrino C (2015). Can early serum lipase measurement be routinely implemented to rule out clinically significant pancreatic fistula after pancreaticoduodenectomy?. Int J Surg.

[9] Angrisani M, Sandini M, Cereda M, Paiella S, Capretti G, Nappo G (2020). Preoperative adiposity at bioimpedance vector analysis improves the ability of Fistula Risk Score (FRS) in predicting pancreatic fistula after pancreatoduodenectomy. Pancreatology.

[10] Lao M, Zhang X, Guo C, Chen W, Zhang Q, Ma T (2019). External validation of alternative fistula risk score (a-FRS) for predicting pancreatic fistula after pancreatoduodenectomy. HPB (Oxford).

[11] Sakamoto T, Yagyu Y, Uchinaka EI, Morimoto M, Hanaki T, Tokuyasu N (2019). Predictive significance of C-reactive Protein-to-albumin ratio for postoperative pancreatic fistula after pancreaticoduodenectomy. Anticancer Res.

[12] Liu Y, Li Y, Wang L, Peng CJ (2018). Predictive value of drain pancreatic amylase concentration for postoperative pancreatic fistula on postoperative day 1 after pancreatic resection: an updated meta-analysis. Medicine (Baltimore).

[13] Maggino L, Malleo G, Bassi C, Allegrini V, Beane JD, Beckman RM (2019). Identification of an optimal cut-off for drain fluid amylase on postoperative day 1 for predicting clinically relevant fistula after distal pancreatectomy: a multi-institutional analysis and external validation. Ann Surg.

[14] Linnemann RJA, Patijn GA, van Rijssen LB, Besselink MG, Mungroop TH, de Hingh IH (2019). The role of abdominal drainage in pancreatic resection - a multicenter validation study for early drain removal. Pancreatology.

[15] Kopljar M, Coklo M, Krstacic A, Krstacic G, Jelec V, Zovak M (2019). Validation of a clinical score in predicting pancreatic fistula after pancreaticoduodenectomy. Acta Chir Belg..

[16] Li Y, Zhou F, Zhu DM, Zhang ZX, Yang J, Yao J (2019). Novel risk scoring system for prediction of pancreatic fistula after pancreaticoduodenectomy. World J Gastroenterol.

[17] Petrova E, Lapshyn H, Bausch D, D'Haese J, Werner J, Klier T (2019). Risk stratification for postoperative pancreatic fistula using the pancreatic surgery registry StuDoQ|Pancreas of the German Society for General and Visceral Surgery. Pancreatology.

[18] Kang JS, Park T, Han Y, Lee S, Kim JR, Kim H (2019). Clinical validation of scoring systems of postoperative pancreatic fistula after pancreatoduodenectomy: applicability to Eastern cohorts?. Hepatobil Surg Nutr.

[19] Teramukai S, Okuda Y, Miyazaki S, Kawamori R, Shirayama M, Teramoto T (2016). Dynamic prediction model and risk assessment chart for cardiovascular disease based on on-treatment blood pressure and baseline risk factors. Hypertension Res.

[20] Li L, Luo S, Hu B, Greene T (2017). Dynamic prediction of renal failure using longitudinal biomarkers in a cohort study of chronic kidney disease. Stat Biosci.

[21] Parast L, Mathews M, Friedberg MW (2019). Dynamic risk prediction for diabetes using biomarker change measurements. BMC Med Res Methodol.

[22] Liu R, Li M, Liu ZP, Wu J, Chen L, Aihara K (2012). Identifying critical transitions and their leading biomolecular networks in complex diseases. Sci Rep.

[23] Yang B, Li M, Tang W, Liu W, Zhang S, Chen L (2018). Dynamic network biomarker indicates pulmonary metastasis at the tipping point of hepatocellular carcinoma. Nat Commun.

[24] Kang CM, Lee JH (2015). Pathophysiology after pancreaticoduodenectomy. World J Gastroenterol.

[25] van Hilst J, Brinkman DJ, de Rooij T, van Dieren S, Gerhards MF, de Hingh IH (2019). The inflammatory response after laparoscopic and open pancreatoduodenectomy and the association with complications in a multicenter randomized controlled trial. HPB (Oxford).

[26] Bassi C, Marchegiani G, Dervenis C, Sarr M, Abu Hilal M, Adham M (2017). The 2016 update of the International Study Group (ISGPS) definition and grading of postoperative pancreatic fistula: 11 Years After. Surgery.

[27] Shinde RS, Acharya R, Chaudhari VA, Bhandare MS, Mungroop TH, Klompmaker S (2020). External validation and comparison of the original, alternative and updated-alternative fistula risk scores for the prediction of postoperative pancreatic fistula after pancreatoduodenectomy. Pancreatology.

[28] Hayashi H, Amaya K, Fujiwara Y, Tokai R, Sugimoto Y, Hashimoto Y (2020). Comparison of three fistula risk scores after pancreatoduodenectomy: a single-institution retrospective study. Asian J Surg..

[29] Torphy RJ, Chapman BC, Friedman C, Nguyen C, Bartsch CG, Meguid C (2019). Quality of life following major laparoscopic or open pancreatic resection. Ann Surg Oncol.

[30] Suresh K, Taylor JMG, Tsodikov A (2019). A Gaussian copula approach for dynamic prediction of survival with a longitudinal biomarker. Biostatistics.

[31] Lee SR, Kim HO, Shin JH (2019). Significance of drain fluid amylase check on day 3 after pancreatectomy. ANZ J Surg.

[32] Pepys MB, Hirschfield GM (2003). C-reactive protein: a critical update. J Clin Invest.

[33] Yin M, Si L, Qin W, Li C, Zhang J, Yang H (2018). Predictive value of serum albumin level for the prognosis of severe sepsis without exogenous human albumin administration: a prospective cohort study. J Intensive Care Med.

[34] Winter JM, Cameron JL, Yeo CJ, Alao B, Lillemoe KD, Campbell KA (2007). Biochemical markers predict morbidity and mortality after pancreaticoduodenectomy. J Am Coll Surg..

[35] Kaplan M, Ates I, Akpinar MY, Yuksel M, Kuzu UB, Kacar S (2017). Predictive value of C-reactive protein/albumin ratio in acute pancreatitis. Hepatobiliary Pancreat Dis Int.

[36] Colley CM, Fleck A, Goode AW, Muller BR, Myers MA (1983). Early time course of the acute phase protein response in man. J Clin Pathol.

[37] Takeda Y, Saiura A, Inoue Y, Mise Y, Ishizawa T, Takahashi Y (2019). Early fistulography can predict whether biochemical leakage develops to clinically relevant postoperative pancreatic fistula. World J Surg..

[38] Yeh DD, Johnson E, Harrison T, Kaafarani HMA, Lee J, Fagenholz P (2018). Serum levels of albumin and prealbumin do not correlate with nutrient delivery in surgical intensive care unit patients. Nutr Clin Pract.

[39] Kim JH, Lee H, Choi HH, Min SK, Lee HK (2019). Nutritional risk factors are associated with postoperative complications after pancreaticoduodenectomy. Ann Surg Treatment Res.

[40] Nagai M, Sho M, Akahori T, Tanaka T, Kinoshita S, Nishiofuku H (2015). Impact of preoperative asymptomatic renal dysfunction on clinical course after pancreatoduodenectomy. J Hepato-Biliary-Pancreatic Sci.

[41] Meersch M, Schmidt C, Zarbock A (2016). Patient with chronic renal failure undergoing surgery. Curr Opin Anaesthesiol.

[42] Reese T, Fard-Aghaie MH, Makridis G, Kantas A, Wagner KC, Malago M (2019). Renal impairment is associated with reduced outcome after associating liver partition and portal vein ligation for staged hepatectomy. J Gastrointest Surg.

[43] Blitz JD, Shoham MH, Fang Y, Narine V, Mehta N, Sharma BS (2016). Preoperative renal insufficiency: underreporting and association with readmission and major postoperative morbidity in an Academic Medical Center. Anesth Analg.

[44] Muscari F, Suc B, Kirzin S, Hay JM, Fourtanier G, Fingerhut A (2006). Risk factors for mortality and intra-abdominal complications after pancreatoduodenectomy: multivariate analysis in 300 patients. Surgery.

[45] Sulpice L, Rayar M, D'Halluin PN, Harnoy Y, Merdrignac A, Bretagne JF (2012). Impact of age over 75 years on outcomes after pancreaticoduodenectomy. J Surg Res.

[46] Fang CH, Chen QS, Yang J, Xiang F, Fang ZS, Zhu W (2016). Body mass index and stump morphology predict an increased incidence of pancreatic fistula after pancreaticoduodenectomy. World J Surg.

[47] Ellis RJ, Brock Hewitt D, Liu JB, Cohen ME, Merkow RP, Bentrem DJ (2019). Preoperative risk evaluation for pancreatic fistula after pancreaticoduodenectomy. J Surg Oncol.

[48] Mathur A, Pitt HA, Marine M, Saxena R, Schmidt CM, Howard TJ (2007). Fatty pancreas: a factor in postoperative pancreatic fistula. Ann Surg.

[49] Kiyochi H, Matsukage S, Nakamura T, Ishida N, Takada Y, Kajiwara S (2015). Pathologic assessment of pancreatic fibrosis for objective prediction of pancreatic fistula and management of prophylactic drain removal after pancreaticoduodenectomy. World J Surg.

[50] Deng Y, Zhao B, Yang M, Li C, Zhang L (2018). Association between the incidence of pancreatic fistula after pancreaticoduodenectomy and the degree of pancreatic fibrosis. J Gastrointest Surg.

[51] Yang YM, Tian XD, Zhuang Y, Wang WM, Wan YL, Huang YT (2005). Risk factors of pancreatic leakage after pancreaticoduodenectomy. World J Gastroenterol.

[52] Frozanpor F, Loizou L, Ansorge C, Lundell L, Albiin N, Segersvard R (2014). Correlation between preoperative imaging and intraoperative risk assessment in the prediction of postoperative pancreatic fistula following pancreatoduodenectomy. World J Surg.

[53] Kim JY, Park JS, Kim JK, Yoon DS (2013). A model for predicting pancreatic leakage after pancreaticoduodenectomy based on the international study group of pancreatic surgery classification. Korean J Hepatobiliary Pancreat Surg.

[54] Yanagimoto H, Satoi S, Yamamoto T, Toyokawa H, Hirooka S, Yui R (2014). Clinical impact of preoperative cholangitis after biliary drainage in patients who undergo pancreaticoduodenectomy on postoperative pancreatic fistula. Am Surg.

[55] Hatano M, Watanabe J, Kushihata F, Tohyama T, Kuroda T, Koizumi M (2015). Quantification of pancreatic stiffness on intraoperative ultrasound elastography and evaluation of its relationship with postoperative pancreatic fistula. Int Surg.

[56] Xia X, Huang C, Cen G, Qiu ZJ (2015). Preoperative diabetes as a protective factor for pancreatic fistula after pancreaticoduodenectomy: a meta-analysis. Hepatobil Pancreat Dis Int.

[57] Mussle B, Oehme F, Schade S, Sommer M, Bogner A, Hempel S (2019). Drain amylase or lipase for the detection of POPF-adding evidence to an ongoing discussion. J Clin Med..

[58] Cai Y, Luo H, Li Y, Gao P, Peng B (2019). A novel technique of pancreaticojejunostomy for laparoscopic pancreaticoduodenectomy. Surg Endosc.

[59] Li T, D'Cruz RT, Lim SY, Shelat VG (2020). Somatostatin analogues and the risk of post-operative pancreatic fistulas after pancreatic resection - a systematic review & meta-analysis. Pancreatology.

[60] Lillemoe KD (2011). Prevention, evaluation, and treatment of leaks after gastrointestinal surgery: prevention of leaks after pancreatic surgery. J Gastrointest Surg.

[61] Dalton EC, Johns MS, Rhodes L, Merritt WT, Petrelli NJ, Tiesi GJ (2020). Meta-analysis on the effect of pasireotide for prevention of postoperative pancreatic fistula. Am Surg.

[62] Sowier A, Pyda P, Sowier S, Kapturzak J, Rybak A, Bialecki J (2020). Postoperative negative-pressure drainage through a PEG tube can prevent pancreatic fistula after pancreatoduodenectomy. Hepatobil Pancr Dis Int.

[63] Laaninen M, Sand J, Nordback I, Vasama K, Laukkarinen J (2016). Perioperative hydrocortisone reduces major complications after pancreaticoduodenectomy: a randomized controlled trial. Ann Surg.

[64] Adachi T, Ono S, Matsushima H, Soyama A, Hidaka M, Takatsuki M (2019). Efficacy of triple-drug therapy to prevent pancreatic fistulas in patients with high drain amylase levels after pancreaticoduodenectomy. J Surg Res.

